# Nanotechnology Approaches in Tackling Cardiovascular Diseases

**DOI:** 10.3390/molecules24102017

**Published:** 2019-05-27

**Authors:** Ray Putra Prajnamitra, Hung-Chih Chen, Chen-Ju Lin, Li-Lun Chen, Patrick Ching-Ho Hsieh

**Affiliations:** Institute of Biomedical Sciences, Academia Sinica, 128 Section 2 Academia Road, Nangang District, Taipei 115, Taiwan; ray.prajnamitra@ibms.sinica.edu.tw (R.P.P.); byronbio@ibms.sinica.edu.tw (H.-C.C.); cjlin@ibms.sinica.edu.tw (C.-J.L.); lilun0817@ibms.sinica.edu.tw (L.-L.C.)

**Keywords:** cardiovascular diseases, drug delivery, nanoparticle, biodistribution, nanogel, nanopatch, nanomaterial, cell sheet

## Abstract

Cardiovascular diseases have continued to remain a leading cause of mortality and morbidity worldwide. Poor proliferation capability of adult cardiomyocytes disables the heart from regenerating new myocardium after a myocardial ischaemia event and therefore weakens the heart in the long term, which may result in heart failure and death. Delivery of cardioprotective therapeutics soon after the event can help to protect the heart from further cell death and improve cardiac function, but delivery methods and potential side effects of these therapeutics may be an issue. Advances in nanotechnology, particularly nanoparticles for drug delivery, have enabled researchers to obtain better drug targeting capability, thus increasing the therapeutic outcome. Detailed study of nanoparticles in vivo is useful as it can provide insight for future treatments. Nanogel can help to create a more favourable environment, not only for a sustained delivery of therapeutics, but also for a better navigation of the therapeutics to the targeted sites. Finally, if the damage to the myocardium is too severe for drug treatment, nanopatch can help to improve cardiac function and healing by becoming a platform for pluripotent stem cell-derived cardiomyocytes to grow for the purpose of cell-based regenerative therapy.

## 1. Introduction

Despite the rapid growth of the global healthcare industry in recent years, cardiovascular diseases (CVDs) have continued to be a prominent cause of mortality and morbidity. Earlier this year the American Heart Association reported that in 2016, CVDs contributed to around 17.6 million deaths worldwide [1]. Among the different types of diseases that fall under the umbrella of CVDs, ischaemic heart disease remains the most common cause of death.

Ischaemia is a condition where an occlusion takes place in the artery, interrupting blood flow and oxygen supply, causing damage to a specific area or tissue in the body. The occlusion can be caused by a clot due to atherosclerosis, thrombosis, or embolism. An ischaemic event that takes place in the heart deprives it of its blood and oxygen supply, resulting in myocardial infarction (MI) or heart attack. When this happens, the most common treatment is to restore the blood flow by relieving the occlusion as soon as possible. Paradoxically, however, the concomitant restoration of blood flow—also known as reperfusion—may inflict additional injury and induce further cell death through the formation of reactive oxygen species. Altogether, this phenomenon is known as the ischaemia/reperfusion (I/R) injury [2].

A heart that suffers from MI may lose around 25% of its cardiomyocytes (CMs) during the episode [3]. Poor proliferation capability of adult CMs renders the heart incapable of regenerating new CMs to replace those it has lost. As a result, it heals itself through the formation of fibrotic scar tissue which, unlike CMs, is not capable of beating. This healing process, known as ventricular remodeling, consequently increases the burden that the heart needs to withstand and therefore, slowly weakens its muscles. The heart will then deteriorate by gradually losing its contractility and muscle strength and, in the long term, this may result in congestive heart failure and ultimately, death.

It is therefore obvious that CMs are vital in maintaining heart function. Cell therapy using CMs derived from embryonic stem cells (ESCs) or induced pluripotent stem cells (iPSCs) provides a unique avenue in the treatment of CVDs, whereby new CMs are regenerated and engrafted on to patients. The progress in stem cell treatment research, however, has not been without its difficulties. Problems such as immunogenicity, slow CM maturation, transplantation issues, and potential formation of teratomas to name a few [3], are actively being addressed by many research groups worldwide. If the damage to the myocardium is not too severe, it is possible for cardioprotective therapeutics to be administered to the patients as an alternative to stem cell treatment, in the hope of protecting CMs from further damage and death following heart injury.

The advent of nanotechnology has revolutionised the field of biomedicine through the development of biocompatible nanomaterials. These nanomaterials have been extensively exploited for various biomedical purposes, including the treatment of CVDs, and have resulted in improved therapeutic benefits overall. Several applications of these nanomaterials include nanoparticles for drug delivery, scaffolds for stem cell modulation, in vivo imaging agents, and platforms for tissue engineering, among others.

In this review, we report the advances in the field to which our lab and others have contributed. We categorise the types of nanomaterials based on the material that they comprise: Nanoparticles, nanogel, and nanopatch. We also show their applications as well as challenges that are hampering the progress, which future research should take into consideration. Altogether, we highlight the considerable role that nanotechnology has played in advancing the treatment of CVDs.

## 2. Nanoparticles

### 2.1. Nanoparticles for the Delivery of Therapeutics

A wide range of therapeutics are available for heart treatment following an ischaemic episode. To ensure the delivery of these therapeutics to the myocardium however, direct intramyocardial injection or intracoronary catheterisation may be required [4]. We previously reported functionalised poly(lactic-co-glycolic acid) (PLGA) nanoparticles carrying insulin-like growth factor (IGF)-1, delivered to the myocardium via intramyocardial injection, which successfully provided cardioprotection to the heart, post-MI in mice [5]. Even though intramyocardial injection showed promising results, it is a very invasive technique and risks inflicting further damage to the heart. On the other hand, intracoronary catheterisation may not be suitable for some therapeutics and may risk inducing embolisation [4]. All of these reasons make intravenous delivery a better choice as it is less invasive and less risky.

When therapeutics are administered through systemic intravenous injection, they will go along the circulatory system. Taking advantage of this phenomena, we reported a successful prevention of thrombus formation in an acute hind limb thromboembolism murine model, using heparin-conjugated carbon nanocapsules delivered by intravenous injection [6]. Carbon nanocapsules were opted as they are more biocompatible in comparison to other carbon-based nanomaterials and more suitable for intravenous delivery [6,7]. Heparin-conjugated carbon nanocapsules were shown to display better antithrombotic activity and extend the formation time of the thrombus longer, compared to heparin or carbon nanocapsules alone [6].

As therapeutics go along the circulatory system after intravenous injection, they are taken up by various organs, retained, and ultimately cleared. Non-functionalised therapeutics are not capable of targeting the heart specifically and will mostly end up in reticuloendothelial organs, such as the liver and spleen [4]. Therefore, it is now common to “protect” the therapeutics by encapsulating them inside nanoparticles. Upon encapsulation, these therapeutics may have a different retention profile compared to their free form, depending on the physicochemical characteristics of the carriers. Drug encapsulation in nanoparticles can be advantageous as it can increase the retention and circulatory half-lives of the drugs [4,5,8]. The surface of these nanoparticles can also be modified with various functional groups that enable active targeting of nanoparticles to certain sites, cells, or tissues. A common example of surface customisation is the attachment of polyethylene glycol (PEG), also known as PEGylation. Nanoparticles that are PEGylated have been reported to possess increased stability and circulatory half-life as well as reduced risk of aggregation [4,8,9]. Surface customisation can also be performed using peptides [10], proteins [11], aptamers [12,13], or small molecules [14,15], which may enable the active targeting of the nanoparticles.

**Figure 1:**
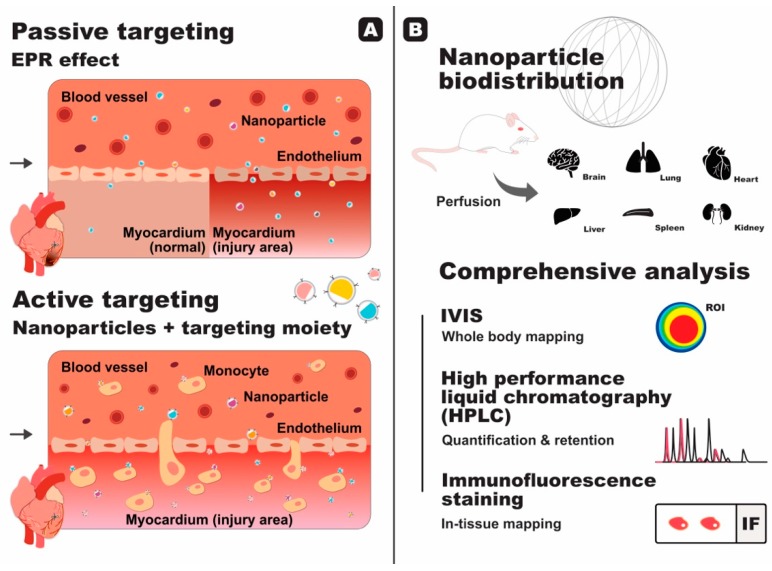
The use of functionalised nanoparticles for the treatment of cardiac ischaemic injury: (**A**) Passive targeting via the enhanced permeability and retention (EPR) effect in comparison to active targeting, using nanoparticles decorated with targeting moieties. Nanoparticles bearing monocyte-targeting moiety are able to actively attach onto the surface of monocytes and extravasate into the injured area; (**B**) Nanoparticles biodistribution study is indispensable in order to investigate the fate of nanoparticles in vivo. Perfusion is performed to rid the organs of freely circulating nanoparticles in the blood prior to organ collection. Overall, biodistribution study maps out the distribution of nanoparticles throughout the body and within the tissue. It also shows the amount retained in each tissue, providing researchers with a complete overview of the fate of their nanoparticles.

Nanoparticles without active targeting moieties are still capable of reaching diseased sites by passive targeting. The so-called enhanced permeability and retention (EPR) effect has been implicated as the reason behind passive targeting, whereby the vasculature of diseased or injured sites gradually become more permeable, enabling drug-containing nanoparticles to permeate through (Figure 1A). The EPR effect has been utilised for tumour targeting because it is persistent for a long duration [16,17]. The production of vascular permeability factor [18] as well as several compounds that enhance permeability compounds, such as nitric oxide, peroxynitrite, and bradykinin [19], have been linked to the increase of permeability of the diseased vasculature. On the other hand, the absence of lymphatic vessels in tumours [19,20] and the unidirectional nature of extravasation through this vasculature [21] are thought to be the main causes of nanoparticle retention and accumulation in tumour sites.

The EPR effect in the myocardium after an ischaemic episode has also been reported [22]. The upregulation of vascular endothelial growth factor (VEGF) production takes place after MI to promote angiogenesis and restore oxygen supply to the damaged myocardium [23,24]. Furthermore, VEGF is also known to increase vascular permeability [23,25,26], therefore resulting in the post-MI EPR effect in the myocardium [23]. Contrary to the EPR effect in tumours, however, post-MI EPR effect does not last long and begins to diminish after 24–48 h [11,22] with partial functional recovery observed after two weeks [22]. A long period of treatment is generally required to treat and heal the heart after an ischaemic episode to prevent negative left ventricular remodelling [27]. This time window is therefore too short for the delivery of the necessary therapeutics.

Despite this limitation, several studies have reported the use of the EPR effect for passive targeting of the heart after an ischaemic episode [27,28,29,30]. One of the studies reported the use of nanoparticles that are able to target the heart through the EPR effect. These nanoparticles self-aggregate upon stimulation by matrix metalloproteinase (MMPs)—enzymes that are present and upregulated in MI—in particular, MMP-2 and MMP-9. The aggregation forms a scaffold that is capable of retaining itself for up to 28 days post-MI [27]. Although the study has successfully increased the retention time, delivery of the nanoparticles would still have to be performed before the EPR effect diminishes. Thus, elimination of the reliance on the EPR effect is still the ideal solution. This can be achieved by exploiting nanoparticles decorated with active targeting moieties.

Scott et al. [31] made use of the increased expression of P-selectin within the vasculature that lies between normal and ischaemic myocardium by conjugating liposomes with an anti-P-selectin antibody. VEGF was encapsulated in these liposomes to promote angiogenesis in the infarct zone. The authors reported that delivery of the anti-P-selectin-conjugated liposomes to a rat MI model successfully improved vascularity and cardiac function, as indicated by the improvement of systolic function as well as the increase in fractional shortening [31].

In 2015, we reported a novel strategy to deliver therapeutics to the heart using liposome nanoparticles coated with a platelet membrane protein, which we coined platelet-like proteoliposome (PLP) [11]. Splenic monocytes are continuously recruited to the infarcted heart to aid with heart healing [32,33,34] in an EPR effect-independent manner (Figure 1A). It has also been reported that in MI patients, platelets are able to bind to circulating monocytes [35]. The PLPs were devised to take advantage of the platelet-monocyte binding, mimic this interaction, and use monocytes as a shuttle bus that can deliver the drugs to the heart. Cobalt protoporphyrin (CoPP) was chosen as the drug in this study. CoPP is able to suppress the inflammatory activity of macrophages by inducing heme oxygenase-1 (HO-1) expression, but as a side effect, it is toxic to the liver and kidneys. CoPP-encapsulated PLPs successfully attached themselves to monocytes and piggybacked their way to the infarcted heart without relying on the EPR effect. Upon extravasation to the myocardium, the PLPs were phagocytised by macrophages and the ensuing release of CoPP directly inside the macrophages increased the therapeutic efficacy. Overall, CoPP-encapsulated PLPs displayed enhanced drug targeting capability and successful improvement in cardiac function with minimal side effects, compared to free CoPP or CoPP-encapsulated liposomes alone. Despite the significant increase, however, only around 5% of the injected PLPs were detected in the heart, whilst the majority were trapped in the spleen and liver [11]. Even though this number is higher than what other research had reported, improvement should be made so that a higher number of nanoparticles are able to reach the heart and therefore increase the therapeutic outcome.

### 2.2. Biodistribution Study of Nanoparticles In Vivo

Research involving the study of nanoparticles in vivo usually includes a biodistribution study to investigate the fate of the nanoparticles in the body and to map out in which organs they are taken up. The biodistribution study is even more important when the nanoparticles contain active targeting moieties, in order to prove their targeting capability (Figure 1B).

Using fluorescent nanoparticles or nanoparticles conjugated to a fluorochrome, our lab has routinely used an in vivo imaging system (IVIS) to obtain biodistribution profiles under various physiological conditions [4,36,37]. The advantage of IVIS is that a whole-body analysis can be performed without sacrificing the animals, however, absolute quantification using IVIS is not recommended as signal saturation and tissue autofluorescence can make the quantification inaccurate [36]. Therefore, we routinely employ high performance liquid chromatography (HPLC) for absolute quantification purposes, due to its superior sensitivity and, more importantly, because it also involves extraction and separation of the fluorescent dye from the tissue. We also make use of immunofluorescence staining in order to further confirm and analyse the distribution within the cells or tissues [4,36,37].

Biodistribution studies are not limited to using fluorescent-based techniques as radiolabeled nanoparticles can be and have been used to assess biodistribution profiles [8,38]. Lameijer et al. [38] reported nanoparticles constructed from high density lipoprotein (HDL) human apolipoprotein A-I that encapsulated a small molecule inhibitor for tumour necrosis factor receptor-associated factor 6 (TRAF6). This treatment managed to rapidly reduce plaque inflammation in transgenic apolipoprotein E-deficient (*Apoe*^−/−^) atherosclerotic mice within one week of therapy. The biodistribution study in this research was carried out in murine and non-human primate models, using positron-emission tomography with magnetic resonance imaging (PET/MRI) [38].

To further highlight the importance of biodistribution studies, we previously reported the size-dependent effect of nanoparticle distribution in mice post-I/R injury. Various sizes of PEGylated polystyrene nanoparticles—with core diameters of 20, 100, 200, 500, 1000, and 2000 nm—without active targeting moiety were administered via intravenous injection. Through HPLC, we found that the retention of 20, 100, 200, and 500 nm nanoparticles were significantly higher in I/R-injured hearts compared to sham hearts and no significant difference was found for the retention of 1000 and 2000 nm nanoparticles. Interestingly, immunofluorescence staining gave a more detailed insight into the distribution in the tissue level (Table 1), where 200 and 500 nm nanoparticles appeared to be entrapped in the blood vessel, indicative of the inability to extravasate into the myocardium due to their size [4]. The staining results also showed that there was a preferential accumulation of nanoparticles in the left ventricle (infarct area) compared to the right ventricle (healthy area), thereby underlining the role of the EPR effect in passive targeting. These findings suggest that the diameter of nanoparticles should ideally be kept below 100 nm in order to maximise the efficacy of passive targeting of I/R-injured hearts [4].

## 3. Nanogel

Protein and cell therapies have been considered as promising approaches for treating cardiovascular diseases. They are designed to cure the disease with direct tissue repair and regeneration, while interventions currently adopted only prevent further damage to the injured site. Clinical trials involving protein or cell therapy for the treatment of cardiovascular diseases, however, have failed to outperform the existing therapies. This is mainly caused by the poor retention of the active elements in the injured site. To solve this problem, nanomaterials, such as self-assembling peptide nanofibers [39], hyaluronic acid hydrogels [40], and alginate hydrogels [41], have been developed to serve as scaffolds for capturing therapeutics and maintaining an environment that favours cardiac repair.

**Figure 2:**
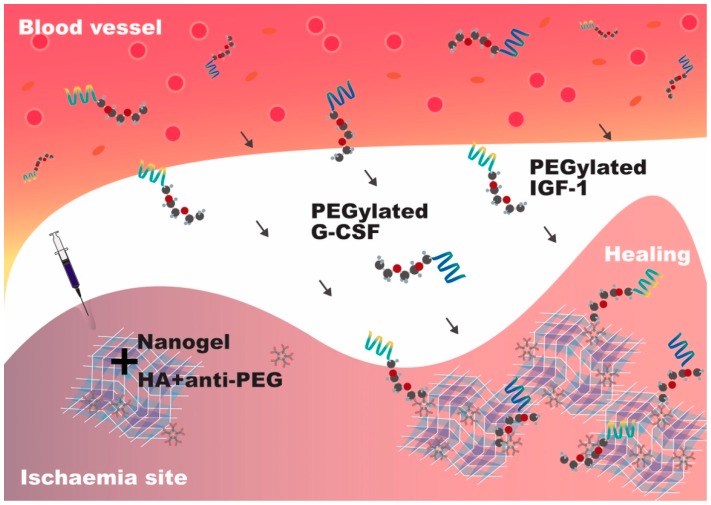
Reloadable multidrug capturing system. The nanogel containing anti-polyethylene glycol (PEG) antibody forms a scaffold which is capable of capturing multiple PEGylated factors from the blood vessel. PEGylated insulin-like growth factor (IGF)-1 is captured and retained, which reduces muscle cell death. Even after capturing the first factor, the reloadable scaffold is still capable of capturing and retaining the second factor, which is PEGylated granulocyte colony-stimulating factor (G-CSF), thereby promoting angiogenesis.

In 2016, our group reported the development of a reloadable multidrug capturing system with anti-polyethylene glycol (PEG) antibody-containing hyaluronic acid (HA) hydrogels as a scaffold to capture PEGylated drugs (Figure 2). Using murine and porcine hind limb ischaemia models, we injected the anti-PEG–HA gel into the ischemic area, where it forms a network capable of capturing and retaining PEGylated IGF-1 and granulocyte colony-stimulating factor (G-CSF) that were intravenously injected successively. Treatment using both factors successfully reduced muscle cell death and promoted angiogenesis, respectively [40].

Using another disease model, we reported the use of a mixture of self-assembling and degradable peptide nanofibers and autologous bone marrow mononuclear cells to treat pigs with induced MI and found the treatment to be efficacious in enhancing both systolic and diastolic functions after injury [39]. We also proved that a mixture of nanofibers and vascular endothelial growth factors promotes arteriogenesis, which results in cardiac systolic function improvement and infarct size reduction within four weeks after MI in both murine and porcine models [42]. The biodegradable peptide nanofibers sustained the damaged site for three months and provided an advantageous microenvironment for cardiac repair as well as mechanical strength. It should be noted that the efficacy of cell therapy is time-dependent, as the treatment should be adopted within four days post-MI [43]. In summary, these studies demonstrated that nanofibers and nanogels are crucial in enhancing the efficacy of cell/protein therapy and restoring heart function in the long term.

## 4. Nanopatch

If the damage to the myocardium is very severe, only relying on drug treatment may not be sufficient and therefore transplantation or stem cell therapy can be opted. ESC- and iPSC-derived CMs are promising cell sources to repair the injured heart after MI. Direct injection of murine ESCs or human iPSCs (hiPSCs) to post-MI rat hearts has been shown to improve heart functions, however, long-term retention, survival, and integration of the implanted CMs are some of the major issues that need to be addressed [44,45]. To overcome these issues, bioengineered cardiac grafts have been developed to aid with post-MI heart repair.

Sekine et al. [46,47] cultured neonatal rat CMs on their own or along with endothelial cells in temperature-responsive culture dishes to generate three-dimensional cell sheets. They transplanted the cell sheets into the infarct heart of a rat MI model and demonstrated improved long-term retention and survival of the transplanted CMs in the infarct area [46,47]. The left ventricular dysfunction can be assessed by decreased left ventricle wall thickness and fractional shortening, which represents the percentage change in the left ventricular cavity during systolic contraction. As a result, the hearts receiving cell sheet transplantation had improved fractional shortening and left ventricle wall thickness, as well as neovascularisation [46]. Using the same temperature-responsive cell sheet technology, Kawamura et al. [48] prepared hiPSC-derived CM sheets, combined with the omental flap, and transplanted the cell sheets into a porcine ischemic cardiomyopathy model. The omental flap was derived from the peritoneal layers and served as a blood supply for the cell sheets. Similarly, they demonstrated that the transplanted hiPSC-derived CMs displayed a better long-term survival on the cell sheets and significantly improved cardiac functions, such as the left ventricle ejection fraction and end diastolic and systolic volumes, in the post-MI porcine heart [48].

**Figure 3:**
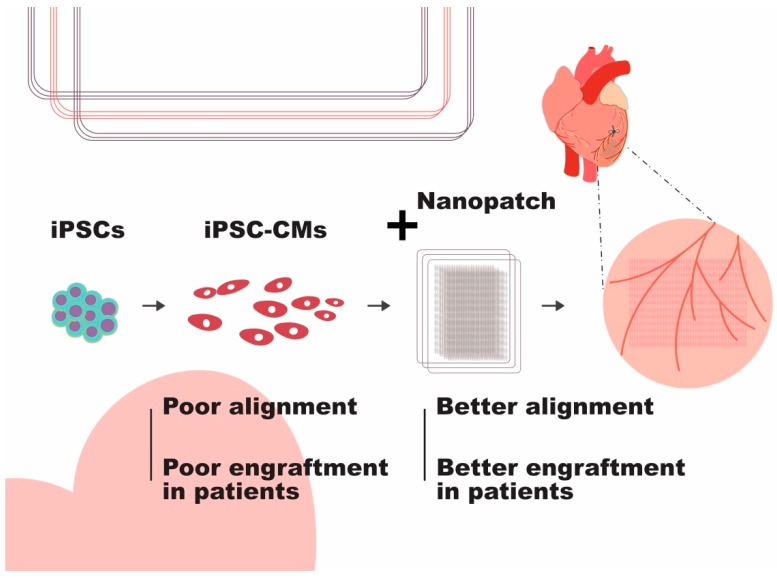
Engraftment of pluripotent stem cell-derived cardiomyocytes can be improved by attachment to a cell sheet. On their own, poor alignment and engraftment are the main issues. However, attaching them to a nanopatch results in better cardiomyocyte alignment and, ultimately, better engraftment in patients.

Apart from using cells alone, we developed an aligned-orientated nanofibrous electrospun patch that serves as a three-dimensional scaffold to align the injected rat neonatal CMs and endothelial cells [49]. Electrospinning techniques allow us to generate polyacrylonitrile nanofibers of 320 ± 32 nm in diameter and align the nanofibers into patches with a variation in fibre orientation of less than 10 degrees. Unlike the cell sheets alone, the nanofibrous patch itself is capable of providing the injured heart with some mechanical support. Moreover, rats with the cell-seeded cardiac patch implantation at two months post-MI displayed reductions in infarct size and fibrosis, and improvement in cardiac function and cardiac hemodynamic parameters. It is worth noting that the implanted cardiac patch formed a functional vascular connection with the host circulatory system, indicative of an improved blood supply to the cardiac patch and thus potential long-term survival of the patch (Figure 3).

Before we can apply the cardiac patch technology for post-MI therapy, however, there are still some obstacles that needed to be overcome. It is important that the cardiac patch is functionally integrated onto the host heart, including vasculature connection to support the implanted cells for long-term survival. The electromechanical coupling of the implanted cardiac patch with the host heart is another issue that should be considered to prevent arrhythmia after transplantation.

## 5. Conclusion and Future Developments

The development of nanomaterials has greatly improved the therapeutic outcome for CVD treatments. Modifications of nanoparticles with active targeting moieties can greatly advance the delivery of cardioprotective drugs to the injured heart with reduced side effects, improving the therapeutic outcome overall. Further studies focusing on active targeting of nanoparticles instead of passive will help to achieve better therapeutics delivery. In addition, detailed biodistribution study helps to provide an insight that can enable better treatment in the future. The use of nanogel can help to create a more favourable environment, not only for sustained delivery of therapeutics, but also for a better navigation of the therapeutics to the target. Cardiac cell sheet provides a novel platform for cell-based therapy. Combined with nanofibrous materials, the cardiac patch can also provide mechanical support to the injured heart. A nanopatch capable of aiding electromechanical coupling of implanted CMs with the host heart would be a major breakthrough in stem cell therapy. Overall, despite the shortcomings, we have shown that nanotechnology plays a very important role in the treatment of CVDs, not only as a medium for drug delivery but also as a platform for cell-based therapy. We believe that nanotechnology, in particular, nanomedicine, is not only an alternative to stem cell treatment, but also a complement and its advancement will be very valuable for both fields.

## Figures and Tables

**Table 1:** Size-dependent effect of nanoparticle biodistribution in mice, post-ischaemia/reperfusion (I/R) injury.

Nanoparticle Size (nm)	HPLC Quantification (I/R vs. Sham)	Observation through Immunofluorescence Staining
20	Significantly higher in I/R-injured hearts (*p* ≤ 0.05)	Sham hearts Sparse, mostly outside blood vessels I/R hearts Accumulation in the injured left ventricle Few in the uninjured right ventricle
100	Significantly higher in I/R-injured hearts (*p* ≤ 0.05)	Sham hearts Very sparse I/R hearts Clear localisation in the injured left ventricle both inside and outside blood vessels Almost none in the uninjured right ventricle
200	Significantly higher in I/R-injured hearts (*p* ≤ 0.05)	Sham hearts Sparse, mostly inside blood vessels I/R hearts Accumulation in the injured left ventricle mostly inside blood vessels Occasionally found in the uninjured right ventricle
500	Significantly higher in I/R-injured hearts (*p* ≤ 0.05)	Sham hearts Distributed all over the left and right ventricles I/R hearts Colocalisation in the injured area with the majority of nanoparticles within the blood vessels
1000	Higher in I/R-injured hearts but not significant (*p* = 0.08)	Sham hearts Found throughout the entire tissue I/R hearts Found throughout the entire tissue Colocalisation observed in the injured left ventricle Consistently found within blood vessels
2000	Higher in I/R-injured hearts but not significant (*p* = 0.24)	Sham hearts Found throughout the entire tissue I/R hearts Found throughout the entire tissue Colocalistion observed in the injured left ventricle Large clusters found entrapped within blood vessels
